# Outcome analysis of Phase I trial patients with metastatic *KRAS* and/or *TP53* mutant non-small cell lung cancer

**DOI:** 10.18632/oncotarget.25947

**Published:** 2018-09-07

**Authors:** Yudong Wang, Zhijie Wang, Sarina Piha-Paul, Filip Janku, Vivek Subbiah, Naiyi Shi, Kenneth Hess, Russell Broaddus, Baoen Shan, Aung Naing, David Hong, Apostolia M. Tsimberidou, Daniel Karp, Charles Lu, Vali Papadimitrakopoulou, John Heymach, Funda Meric-Bernstam, Siqing Fu

**Affiliations:** ^1^ Department of Investigational Cancer Therapeutics, The University of Texas MD Anderson Cancer Center, Houston, TX, USA; ^2^ Department of Biostatistics, The University of Texas MD Anderson Cancer Center, Houston, TX, USA; ^3^ Department of Pathology, The University of Texas MD Anderson Cancer Center, Houston, TX, USA; ^4^ Department of Thoracic Medical Oncology, The University of Texas MD Anderson Cancer Center, Houston, TX, USA; ^5^ Department of Medical Oncology, The Fourth Hospital of Hebei Medical University, Shijiazhuang, Hebei Province, People’s Republic of China; ^6^ Department of Medical Oncology, Cancer Hospital, Chinese Academy of Medical Sciences & Peking Union Medical College, Beijing, People’s Republic of China; ^7^ Department of Cancer Research, The Fourth Hospital of Hebei Medical University, Shijiazhuang, Hebei Province, People’s Republic of China

**Keywords:** KRAS, TP53, Phase I trial, overall survival, non-small cell lung cancer

## Abstract

*KRAS* and *TP53* mutations, which are the most common genetic drivers of tumorigenesis, are still considered undruggable targets. Therefore, we analyzed these genetic aberrations in metastatic non-small cell lung cancer (NSCLC) for the development of potential therapeutics. One hundred eighty-five consecutive patients with metastatic NSCLC in a phase 1 trial center were included. Their genomic aberrations, clinical characteristics, survivals, and phase 1 trial therapies were analyzed. About 10%, 18%, 36%, and 36% of the patients had metastatic *KRAS*+/*TP53*+, *KRAS*+/*TP53*-,*KRAS*-/*TP53*+, and *KRAS*-/*TP53*- NSCLC, respectively. The most common concurrent genetic aberrations beside *KRAS* and/or *TP53* (>5%) were KIT, epidermal growth factor receptor, PIK3CA, c-MET, BRAF, STK11, ATM, CDKN2A, and APC. *KRAS*+/*TP53*+ NSCLC did not respond well to the phase 1 trial therapy and was associated with markedly worse progression-free (PFS) and overall (OS) survivals than the other three groups together. *KRAS* hotspot mutations at locations other than codon G12 were associated with considerably worse OS than those at this codon. Gene aberration-matched therapy produced prolonged PFS and so was anti-angiogenesis in patients with *TP53* mutations. Introduction of the evolutionary action score system of *TP53* missense mutations enabled us to identify a subgroup of NSCLC patients with low-risk mutant p53 proteins having a median OS duration of 64.5 months after initial diagnosis of metastasis. These data suggested that patients with metastatic dual *KRAS+/TP53+* hotspot-mutant NSCLC had poor clinical outcomes. Further analysis identified remarkably prolonged survival in patients with low-risk mutant p53 proteins, which warrants confirmatory studies.

## INTRODUCTION

Non-small cell lung cancer (NSCLC), constituting more than 80% of all lung cancers, is the leading cause of cancer morbidity and mortality worldwide [1]. Mutations of *KRAS*, a member of the RAS family, are among the most common oncogene mutations in NSCLC patients, identified in up to 30% of NSCLC cases [2–5]; are most frequently activating point mutations at codons G12, G13, and Q61 [6]; occur most often in patients with adenocarcinoma, who are white, and who are current or former smokers [7–9]; and are mutually exclusive of epidermal growth factor receptor (EGFR) mutations and ALK and ROS1 rearrangements [8, 10–12]. Inactivation of the *TP53* gene is the most frequent molecular alteration in NSCLCs. Reported incidence rates for *TP53* exon 5-8 mutations in NSCLC patients ranged from 31% to 79%, with mutations occurring most frequently in squamous cell carcinoma cases [13–15]. *TP53* plays many important roles in the prevention and suppression of abnormal cell growth through cell-cycle arrest, apoptosis, senescence, and DNA repair and induction of drug resistance [15, 16]. Although the prognostic or predictive value of *TP53* mutations has been inconclusive in NSCLC cases [17–23], recent studies demonstrated that antiangiogenic-based therapy may be appropriate for the treatment of *TP53*-mutant NSCLC [24–26].

Both *KRAS* and *TP53* mutations are considered undruggable [27]. In the present study, we reviewed the demographic characteristics and clinical outcomes of patients with metastatic NSCLC who were referred to phase 1 trial center at The University of Texas MD Anderson Cancer Center in an effort to determine the impact of *KRAS* and *TP53* mutations on their disease for the development of potential therapeutics.

## RESULTS

### Patient demographics

Of the 185 consecutive patients with metastatic NSCLC referred to phase 1 trials at MD Anderson, 100 (54%) received phase 1 clinical trial therapy. In this cohort, the median ages were 60 years (range, 26-80 years) at initial metastasis diagnosis, and 62 years (range, 27-82 years) at initial phase I clinic visit, respectively. About 28% (*n*=52) of the patients had *KRAS* hotspot mutations, and 47% (*n*=86) had *TP53* hotspot mutations (Table 1). *KRAS* hotspot mutations were significantly more common in current and previous smokers than in never-smokers (33% versus 14%; *p*=0.015), patients with adenocarcinoma than in those with squamous cell carcinoma (32% versus 14%; *p*=0.031), patients who had prior surgery for tumor resection than those who did not (38% versus 22%; *p*=0.029), and patients who had prior EGFR inhibition than in those who did not (35% versus 19%; *p*=0.021). In comparison, *TP53* hotspot mutations were more common in male than in female patients (54% versus 38%; *p*=0.030) patients without adenocarcinoma than in those with adenocarcinoma (67% versus 41%; *p*=0.005), and in patients with poorly differentiated tumors than in those without (61% versus 35%; *p*<0.001). No Asian patients presented with *KRAS* hotspot-mutant NSCLC, whereas 31% of non-Asian patients presented with it (*p*=0.007).

**Table 1:** Patient characteristics per *KRAS* and/or *TP53* hotspot mutation status

Parameters	TotalN = 185 (%)	*KRAS+/TP53+*N = 19 (%)	*KRAS-/TP53+*N = 67 (%)	*KRAS+/TP53-*N = 33 (%)	*KRAS-/TP53-*N = 66 (%)
**Gender**					
Male	96 (52)	11 (58)	41 (61)	16 (49)	28 (42)
Female	89 (48)	8 (42)	26 (39)	17 (51)	38 (58)
**Race**					
White	145 (78)	15 (78)	50 (75)	30 (91)	50 (76)
Asian	16 (9)	0 (0)	11(16)	0 (0)	5 (7)
Black	13 (7)	2 (11)	2 (3)	2 (6)	7 (11)
Hispanic	11 (6)	2 (11)	4 (6)	1 (3)	4 (6)
**Never Smoker**	49 (27)	3 (16)	18 (27)	4 (12)	24 (36)
**Pathology**					
Adenocarcinoma	143 (77)	15 (79)	43 (64)	31 (94)	54 (82)
Squamous cell	29 (16)	3 (16)	16 (24)	1 (3)	9 (14)
Others	13 (7)	1 (5)	8 (12)	1 (3)	3 (4)
**Phase I trial therapy**	100 (54)	13 (68)	32 (48)	17 (52)	38 (58)
**ECOG performance**					
0	2 (1)	1 (5)	0 (0)	0 (0)	1 (2)
1	135 (73)	15 (79)	43 (64)	25 (76)	52 (78)
2	34 (18)	3 (16)	20 (30)	3 (9)	8 (12)
3	14 (8)	0 (0)	4 (6)	5 (15)	5 (8)
**BMI**					
BMI<18.5	20 (11)	1 (5)	7 (10)	2 (6)	10 (15)
18.5≤BMI<24	78 (42)	10 (53)	29 (43)	15 (46)	24 (36)
24≤BMI<27	35 (19)	4 (21)	13 (20)	7 (21)	11 (17)
BMI≥27	52 (28)	4 (21)	18 (27)	9 (27)	21 (32)
Prior Surgery	69 (37)	9 (47)	24 (36)	17 (52)	19 (29)
Prior Radiation	132 (71)	13 (68)	47 (70)	25 (76)	47 (71)
Prior VEGF inhibition	54 (29)	6 (32)	17 (25)	10 (30)	21 (32)
Prior EGFR inhibition	83 (45)	3 (16)	32 (48)	13 (39)	35 (53)
Prior systemic treatment (median number, range): 2 (0 – 8)					

Abbreviations: N, number; +, positive hotspot mutation test; –, negative hotspot mutation test; ECOG, East Collaborative Oncology Group; BMI, body mass index, VEGF, vascular endothelial growth factor, and EGFR, epidermal growth factor receptor.

### Concurrent *KRAS* and *TP53* hotspot mutations

Genomic analysis of the 185 NSCLC patients revealed concurrent *KRAS* and *TP53* hotspot mutations (*KRAS*+/*TP53*+) in 19 patients (10%), *TP53* hotspot mutations only (*KRAS*-/*TP53*+) in 67 patients (36%), *KRAS* hotspot mutations only (*KRAS*+/*TP53*-) in 33 patients (18%), and no hotspot mutations (*KRAS*-/*TP53*-) in 66 patients (36%). Besides *KRAS* and *TP53* hotspot mutations, we identified KIT, EGFR, PIK3CA, c-MET, BRAF, STK11, ATM, CDKN2A, and APC hotspot mutations and/or gene variants in more than 5% of patients with metastatic NSCLC as shown in Table 2. *BRAF* hotspot mutations occurred more frequently in *KRAS*-/*TP53*- NSCLC cases than in *KRAS*+ and/or *TP53*+ cases (*p*=0.006). None of the 52 patients with *KRAS* hotspot mutations had EGFR hotspot mutations, whereas 38 of the 133 patients (29%) without *KRAS* mutations harbored EGFR hotspot mutations (*p*<0.001).

**Table 2:** The concurrent hotspot mutation / gene variant status

Parameters	TotalN = 185 (%)	*KRAS+/TP53+*N = 19 (%)	*KRAS-/TP53+*N = 67 (%)	*KRAS+/TP53-*N = 33 (%)	*KRAS-/TP53-*N = 66 (%)
KIT	58 (30.8)	11 (19)	16 (27.6)	12 (20.7)	19 (32.7)
EGFR	38 (20.5)	0	15 (39.5)	0	23 (60.5)
PIK3CA	27 (14.1)	2 (7.4)	10 (37.1)	6 (22.2)	9 (33.3)
c-MET	22 (11.4)	2 (9.1)	4 (18.2)	3 (13.6)	13 (59.1)
BRAF	16 (8.7)	1 (6.3)	3 (18.7)	1 (6.3)	11 (68.7)
STK11	16 (8.7)	2 (12.5)	4 (25)	4 (25)	6 (37.5)
ATM	15 (8.1)	0	4 (26.7)	6 (40)	5 (33.3)
CDKN2A	12 (6.5)	1 (8.3)	6 (50)	2 (16.7)	3 (25)
APC	10 (5.4)	1 (10)	6 (60)	0	3 (30)
KDR	7 (3.8)	0	2 (28.6)	2 (28.6)	3 (42.8)
CTNNB1	6 (3.2)	0	3 (50)	0	3 (50)
SMO	5 (2.7)	2 (40)	2 (40)	0	1 (20)
FBXW7	5 (2.7)	0	2 (40)	0	3 (60)
ERBB2	4 (2.2)	0	2 (50)	0	2 (50)
ERBB4	4 (2.2)	1 (25)	2 (50)	0	1 (25)
IDH1	4 (2.2)	2 (10.5)	0	1 (3)	1 (1.5)
SMAD4	4 (2.2)	0	2 (50)	0	2 (50)
FGFR3	4 (2.2)	0	4 (50)	0	0
FGFR2	3 (1.6)	0	3 (100)	0	0
AKT1	3 (1.6)	1 (33.3)	1 (33.3)	0	1 (33.3)
JAK3	3 (1.6)	0	1 (33.3)	1 (33.3)	1 (33.3)
NOTCH1	3 (1.6)	0	1 (33.3)	2 (66.7)	0
PDGFRA	3 (1.6)	0	2 (66.7)	0	1 (33.3)
FGFR1	2 (1.1)	1 (50)	0	0	1 (50)
GNAS	2 (1.1)	0	0	1 (50)	1 (50)
NRAS	2 (1.1)	0	2 (100)	0	0
ABL1	2 (1.1)	1 (50)	0	0	1 (50)
RET^*^	2 (1.1)	0	2 (100)	0	0
ALK^*^	1 (0.5)	0	0	1 (100)	0
HNF1A	1 (0.5)	1 (100)	0	0	0
MLH1	1 (0.5)	0	1 (100)	0	0
RB1	1 (0.5)	0	0	0	1 (100)

Abbreviations: N, number; +, positive hotspot mutation test; –, negative hotspot mutation test; and ^*^ indicates rearrangement of the gene.

### The impact of *KRAS* and *TP53* hotspot mutations on survivals

The median OS for these 185 patients were 8.9 months (95% confidence interval [CI], 15.7-23.4 months) from initial phase I clinic visit, and 25.9 months (95% CI, 14.3-41.5 months) from initial metastasis diagnosis, respectively. Our results showed that the median OS from initial phase I clinic visit was significantly longer in patients who had good RMH prognostic scores of 0-1 (n=138; 10.7 months, 95%CI, 8.3-13.2 months) than those who had poor RMH prognostic scores of 2-3 (n=47; 4.6 months, 95%CI, 3.1-6.2 months; *p*<0.001). The MDACC prognostic score system was also validated in these patients: scores of 0-1 (n=121; 11.5 months, 95%CI, 9.1-14 months), scores of 2 (n=45; 5 months, 95%CI, 3.7-6.4 months), and scores of >2 (n=19; 1.8 months, 95%CI, 1-2.6 months; *p*<0.001), respectively.

**Figure 1:**
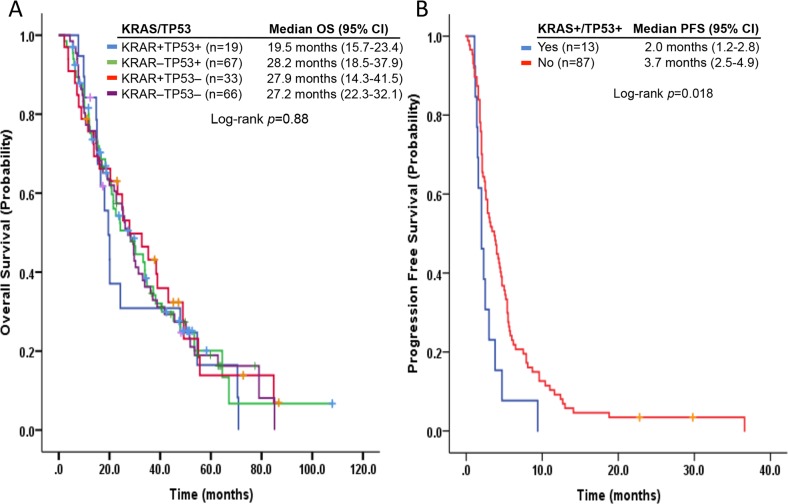
Kaplan-Meier curves of **(A)** overall survivals in patients with metastatic NSCLC according to *KRAS* and *TP53* hotspot mutation status and **(B)** progression-free survivals in patients with metastatic *KRAS+*/*TP53+* NSCLC who received phase 1 trial therapy as the first-line therapy versus those without *KRAS+*/*TP53+* NSCLC.

Survival analysis demonstrated that 19 patients with metastatic *KRAS*+/*TP53*+ NSCLC had a median OS of 19.5 months (95% CI, 15.7-23.4 months) from initial metastasis diagnosis and 7 months (95% CI, 2.1-12 months) from initial phase I clinic visit. In comparison, the median OS were 27.9 months (95% CI, 14.3-41.5 months) and 8.5 months (95% CI, 3.8-13.1 months) in *KRAS*+/*TP53*-, 28.2 months (95% CI, 18.5-37.9 months) and 8.9 months (95% CI, 4.6-13.3 months) in *KRAS*-/*TP53*+, and 27.2 months (95% CI, 22.3-32.1 months) and 9.3 months (95% CI, 4.9-13.6 months) in *KRAS*-/*TP53*- cases, respectively (*p*=0.88, and *p*=0.81) (Figure 1A). Furthermore, the 52 patients with *KRAS* hotspot-mutant NSCLC had a median OS of 24.3 months (95% CI, 15.9-32.7 months) from initial metastasis diagnosis and 7.2 months (95% CI, 3.3-11.2 months) from initial phase I clinic visit, whereas those without *KRAS* mutations had a median OS of 27.2 months (95% CI, 22.3-32.1 months; *p*=0.9) and 9.2 months (95% CI, 5.9-12.5 months; *p*=0.48). Similarly, patients with *TP53* hotspot mutations had a median OS of 24.1 months (95% CI, 16.7-31.6 months) and 7.7 months (95% CI, 3.9-11.5 months), whereas those without *TP53* mutations had 27.9 months (95% CI, 23.5-32.2 months; *p*=0.7) and 9.2 months (95% CI, 5.5-12.8 months; *p*=0.67), respectively.

**Figure 2:**
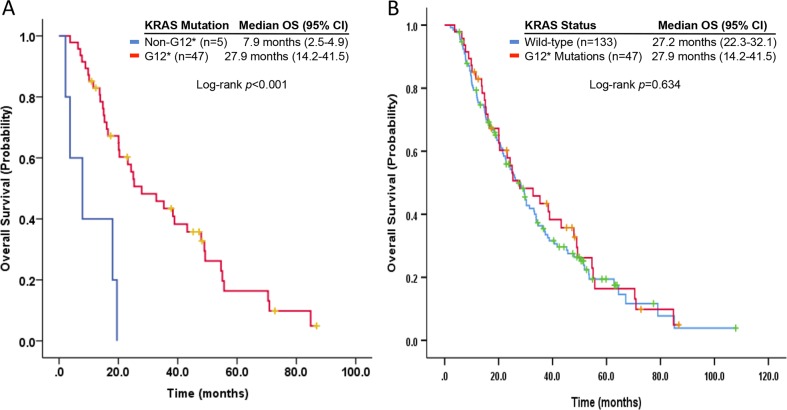
Kaplan-Meier curves of overall survivals in **(A)** patients with metastatic *KRAS* hotspot-mutant NSCLC (codon G12 versus non-G12) and **(B)** patients with *KRAS* hotspot mutations at codon G12 versus those without *KRAS* hotspot mutations.

We identified *KRAS* hotspot mutations at codons G12 (*n* = 47; 90%), G13 (*n*=2; 4%), Q61 (*n*=2; 4%), and I36 (*n*=1; 2%). Patients with metastatic NSCLC harboring *KRAS* hotspot mutations at codon G12 and those without *KRAS* hotspot mutations had similar OS (27.9 months [95% CI, 14.2-41.5 months] versus 27.2 months [95% CI, 22.3-32.1 months]; *p*=0.63) from initial metastasis diagnosis and (9.2 months [95% CI, 5.9-12.5 months] versus 8.5 months [95% CI, 4.2-12.8 months]; *p*=0.73) from initial phase I clinic visit. The two groups combined had a longer median OS than those with *KRAS* hotspot mutations at locations other than codon 12 (7.9 months [95% CI, 0-17 months]; *p*<0.001) (Figure 2), and (4.4 months [95% CI, 0-9.9 months]; *p*=0.073), respectively.

**Figure 3:**
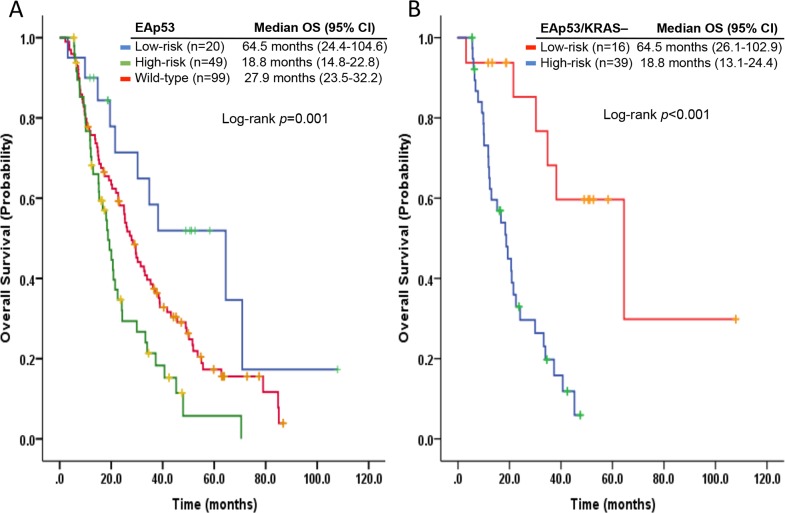
Kaplan-Meier curves of overall survivals according to the EAp53 scores in all patients **(A)** and in those without *KRAS* hotspot mutations **(B)**.

We detected 67 different types of *TP53* hotspot mutations, including missense, nonsense, and frameshift mutations in 86 patients. In 69 patients with *TP53* hotspot missense mutations, two classes of patients were identified: high-risk EAp53 prognostic scores (EAp53-HR; *n*=49) and low-risk EAp53 prognostic scores (EAp53-LR; *n*=20), according to the calculated EAp53 scores. We observed a significantly longer median OS from initial metastasis diagnosis in EAp53-LR patients (64.5 months; 95% CI, 24.4-104.6 months) than in those with EAp53-HR patients (18.8 months; 95% CI, 14.8-22.8 months; *p*=0.001) with a hazard ration (HR) of 0.3 (95% CI, 0.14-0.63; *p*=0.001) after adjustment with ECOG functional status, number of metastatic sites, and serum levels of lactate dehydrogenase and albumin; and in patients without *TP53* hotspot mutations (*n*=99; 27.9 months; 95% CI, 23.5-32.2 months; *p*=0.043) with a HR of 0.52 (95% CI, 0.27-0.99; *p*=0.049) (Figure 3A). Similarly, a significant longer median OS from initial phase I clinic visit was observed in EAp53-LR patients (32 months; 95% CI, 13.1-50.8 months) than in EAp53-HR patients (5.4 months; 95% CI, 4.1-6.8 months; *p*=0.001) with a HR of 0.29 (95% CI, 0.13-0.63; *p*=0.002), and in patients without *TP53* hotspot mutations (9.2 months; 95% CI, 5.5-12.8 months; *p*=0.042) with a HR of 0.51 (95%CI, 0.26-0.98; *p*=0.048).

In patients with concurrent *KRAS* hotspot mutations, we did not observe an OS difference from initial metastasis diagnosis between EAp53-LR (14.8 months; 95% CI, 5.3-24.3 months) and EAp53-HR patients (20.1 months; 95% CI, 14.1-26.1 months; *p*=0.9) with a HR of 0.92 (95%CI, 0.24-3.5; *p*=0.9); and from initial phase I clinic visit between EAp53-LR (6.5 months; 95% CI, 0-21.3 months) and EAp53-HR patients (7 months; 95% CI, 0.1-14 months; *p*=0.55) with a HR of 0.67 (95%CI, 0.17-2.5; *p*=0.55). In patients without detected *KRAS* hotspot mutations, EAp53-LR patients had a significantly longer median OS from initial metastasis diagnosis (64.5 months; 95% CI, 26.1-102.9 months), and from initial phase I clinic visit (32 months; 95% CI, 20.8-43.1 months) than those in EAp53-HR patients (18.8 months; 95% CI, 13.1-24.4 months; *p*<0.001), and (5.4 months; 95% CI, 3.4-7.5 months; *p*=0.001) with a HR of 0.2 (95% CI, 0.07-0.51, *p*=0.001), and 0.23 (95% CI, 0.08-0.6, *p*=0.003), respectively (Figure 3B).

### The impact of phase 1 trial therapy on PFS

**Figure 4:**
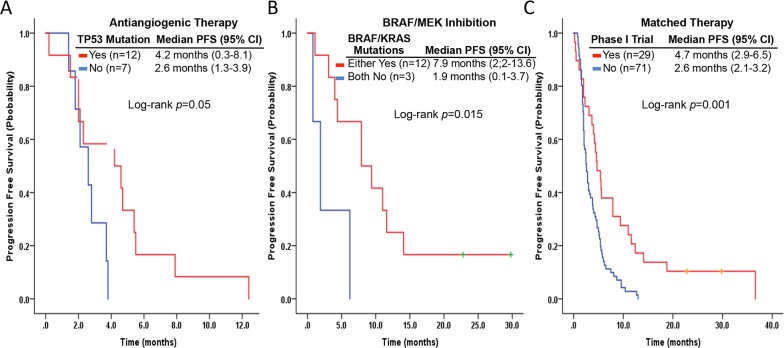
Kaplan-Meier curves of progression-free survivals in patients with metastatic NSCLC who received antiangiogenic phase 1 trial therapy according to *TP53* hotspot mutation status **(A)**, BRAF/MEK inhibitor-based phase 1 trial therapy according to BRAF or *KRAS* hotspot mutation status **(B)**, and matched phase 1 trial therapy versus those who did not **(C)**.

The first-line phase 1 trial therapy led to similar median PFS in *KRAS*+ (n*=*30; 2.3 months; 95% CI, 1.5-3.1 months) and *KRAS*- (*n*=70; 3.4 months; 95% CI, 2.3-4.5 months) patients (*p*=0.15), as well as in *TP53*+ (*n*=45; 2.5 months; 95% CI, 1.6-3.4 months) and *TP53*- (*n*=55; 3.4 months; 95% CI, 2.3-4.5 months) patients (*p*=0.3). *KRAS*+/*TP53*+ patients (*n*=13) had a median PFS of 2 months (95% CI, 1.2-2.8 months), which was significantly shorter than that in *KRAS*+/*TP53*-, *KRAS*-/*TP53*+, and *KRAS*-/*TP53*- patients combined (*n*=87; 3.7 months; 95% CI, 2.5-4.9 months; *p*=0.018) (Figure 1B). Among patients receiving antiangiogenic agent-based phase 1 trial therapy, we observed one partial response and six cases of stable disease (clinical benefit, 58.3%) in *TP53*+ patients (*n*=12). Also, the *TP53*+ patients had a median PFS of 4.2 months (95% CI, 0.3-8.1 months), which was significantly better than that in *TP53*- patients (*n*=7) on clinical benefit (0%; *p*=0.017), and median PFS (2.6 months; 95% CI, 1.3-3.9 months; *p*=0.05) (Figure 4A). In patients who received BRAF and/or MEK inhibitor-based phase 1 trial therapy (*n*=15), we observed four partial responses and six cases of stable disease (clinical benefit, 83.3%) in 12 BRAF+ or *KRAS*+ patients. Also, these patients had a median PFS of 7.9 months (95% CI, 2.2-13.6 months). In contrast, in three BRAF- and *KRAS*- patients, we observed one case of stable disease (clinical benefit, 33.3%; *p*=0.08), and a median PFS of 1.9 months (95% CI, 0.2-3.7 months; *p*=0.015) (Figure 4B).

Including *TP53*+ patients who received antiangiogenic therapy (*n*=12), 29 patients received matched phase 1 trial therapy: BRAF inhibition for BRAF (*n*=9), EGFR inhibition for EGFR (*n*=5: 1 receiving combination therapy with antiangiogenic therapy), MEK inhibitors for *KRAS* (*n*=3), and anti-HER2 inhibition for ERBB2 (*n*=1), leading to 1 complete response, 6 partial responses, and 12 cases of stable disease (clinical benefit, 65.5%). In addition, these patients had a median PFS duration of 4.7 months (95% CI, 2.9-6.5 months), which was significantly better than that in patients who did not receive matched therapy (*n*=71): 1 had a partial response and 22 had stable disease (clinical benefit, 32.4%; *p*=0.002), and their median PFS duration was 2.6 months (95% CI, 2.1-3.2 months; *p*=0.001) (Figure 4C).

## DISCUSSION

We previously reported on an outcome analysis of patients with metastatic *KRAS* and *TP53* hotspot-mutant solid tumors in a phase 1 trial center [28]. In the present study, we focused on the impact of *KRAS* and/or *TP53* hotspot mutations on patients with metastatic NSCLC as well as the impact of phase 1 clinical trial therapy on their survival. We selected 185 consecutive patients from June 2011 to December 2016. We found that the most frequent genomic aberrations in these NSCLC patients were *TP53* (47%), KIT (31%), *KRAS* (28%), EGFR (21%), PIK3CA (14%), c-MET (11%), BRAF (9%), STK11 (9%), ATM (8%), CDKN2A (7%), and APC (5%) mutations, which were similar to previously reported genomic aberrations [29, 30]. About 10% of the patients had *KRAS*+/*TP53*+ NSCLC, whereas 18%, 36%, and 36% had *KRAS*+/*TP53*-, *KRAS*-/*TP53*+, and *KRAS*-/*TP53*- NSCLC, respectively. We identified *KRAS* hotspot mutations at codon G12 (90%) in 47 patients, and 67 different types of *TP53* hotspot mutations, including missense, nonsense, and frameshift mutations in 86 patients. EGFR and *KRAS* hotspot mutations occurred exclusively, whereas we observed BRAF hotspot mutations more frequently in *KRAS*-/*TP53*- patients than in *KRAS*+ and/or *TP53*+ patients as described previously [31–34].

Further analyses produced several interesting findings. First, patients with metastatic *KRAS*+/*TP53*+ NSCLC tended to have poor outcomes with a median OS of 19.5 months (95% CI, 15.7-23.4 months), about 6.8 months shorter than those with metastatic *KRAS*- and/or *TP53*- NSCLC. Consistent with this, patients with metastatic *KRAS*+/*TP53*+ NSCLC did not respond to the phase 1 trial therapy well, with a median PFS of 2 months (95% CI, 1.2-2.8 months), which was significantly worse than that in *KRAS*+/*TP53*-, *KRAS*-/*TP53*+, and *KRAS*-/*TP53*- patients (3.7 months; 95% CI, 2.5-4.9 months). These data imply that the status of KRAS and TP53 mutation in patients with metastatic NSCLC might serve as a prognostic and predictive factor. Further exploration of the concurrent mutational profiling as demonstrated in Table 2 in the setting of KRAS and/or TP53 mutation is warranted to establish their roles to predict prognosis and response in large cohorts of patients. It was noted that few patients from this retrospective cohort were enrolled in phase 1 clinical trials of immunotherapy. Thus, this finding does not apply to future patients, especially when a majority of patients are enrolled in immunotherapy-based phase 1 clinical trials.

Additionally, patients with *KRAS* hotspot mutations at locations other than codon G12 had significantly worse OS than did patients with *KRAS* hotspot mutations at codon G12 and *KRAS*- patients. This finding is consistent with our and others’ previous findings that patients harboring mutations at codon G13 had significantly worse OS than did those without mutations at this codon [28, 35, 36]. This finding may enhance future drug development targeting *KRAS* mutations to differentiate subgroups of *KRAS*-mutant NSCLC. The presence of *KRAS* mutations may influence the efficacy of therapy directed toward other concurrent targets or serve as a prognostic factor for survival.

Use of the evolutionary action score system EAp53 further classified *TP53* missense mutations [37, 38]. We demonstrated that the presence of low-risk *TP53* mutations (EAp53-LR) was associated with significantly better OS than was that of high-risk mutations (EAp53-HR) and the absence of *TP53* hotspot mutations. Of note is that patients with metastatic EAp53-LR NSCLC had a median OS longer than 5 years, indicating that the probability of dying (hazard) was reduced by 70% compared with those with metastatic EAp53-HR NSCLC (HR=0.3; 95% CI, 0.14-0.63; p=0.001), and by 48% compared with those without *TP53* hotspot mutations (HR=0.52; 95% CI, 0.27-0.99; p=0.049). Both results were statistically significant. If this finding is confirmed in future studies, it will differentiate the concurrent consensus that *TP53* mutations are associated with poor clinical outcomes in patients with metastatic NSCLC [23, 39–41]. However, the presence of *KRAS* hotspot mutations made this survival advantage disappear in our study, suggesting the importance of concurrent mutations or genomic profiles to predicting outcomes. To the best of our knowledge, the present retrospective study is the first study of the association between *TP53* mutations and survival in NSCLC patients using the EAp53 system. Future prospective large studies using this system are warranted.

Furthermore, this study demonstrated markedly greater clinical benefit and PFS with matched phase I trial therapy than with phase I trial therapy not targeting genomic aberrations. Specifically, we observed clinical benefit and PFS advantages of matched phase I clinical trial therapy targeting EGFR, BRAF, and ERBB2. However, the difference in OS between the patients who received matched and unmatched therapy was not significant, suggesting that actionable mutations are predictive factors for metastatic NSCLC and/or that more effective novel therapeutic strategies become available to these patients.

We examined potential matched therapy in patients with *KRAS* or *TP53* hotspot mutations. Despite the limited number of patients receiving MEK inhibitor-based therapy, those with metastatic *KRAS*+ NSCLC tended to have better responses than did those with metastatic *KRAS*- NSCLC. In patients with metastatic *TP53* hotspot-mutant NSCLC, antiangiogenic therapy provided significantly better clinical benefit and PFS than those with *TP53-* NSCLC, supporting the concept that *TP53* mutations induce tumor angiogenesis [24, 25, 42–44]. Further prospective studies are warranted to determine whether antiangiogenic therapy can be administered as matched therapy for metastatic NSCLC in patients with *TP53* hotspot mutations, especially those EAp53-HR patients with a high EAp53 score.

Our study had some limitations. Unknown biases and patient selection influenced our analyses because this was a retrospective, single-center chart review. Also, the small number of patients limited confirmatory analyses, especially in subgroup studies. The low frequency of metastatic *KRAS*/*TP53* hotspot-mutant NSCLC in this cohort may not reflect the real picture of NSCLC patients in society, as physicians requested genomic profiling only for patients with sufficient bone marrow, liver, and kidney function as well as decent performance function (about three quarters of the patients had an Eastern Cooperative Oncology Group score of 1 or better). Furthermore, concurrent *KRAS* and *TP53* mutations produce potentially synergistic biological effects. NSCLC associated with both *KRAS* and *TP53* mutations may present as a unique cancer subtype with distinct and aggressive biological behavior [45–49], resulting in many patients with metastatic *KRAS*+/*TP53*+ NSCLC not being selected for genomic profiling owing to poor functional status and organ dysfunction. We have to accept that this manuscript presents a limited set of retrospective data, which can only lead to preliminary hypotheses for future studies. Therefore, larger prospective population studies are required to further define the impact of *KRAS* and/or *TP53* mutations on patients with metastatic NSCLC.

In conclusion, our study showed that *KRAS* (28%) and *TP53* (47%) hotspot mutations occurred frequently in patients with metastatic NSCLC. *KRAS* hotspot mutations were more common in non-Asian patients than in Asian ones, previous and current smokers than in never-smokers, and patients without EGFR and BRAF mutations than in patients with EGFR and BRAF mutations; whereas *TP53* hotspot mutations were more common in male patients than in female ones, patients with squamous cell carcinoma than in those with adenocarcinoma, and patients with poorly differentiated tumors than in well differentiated tumors. Patients with metastatic *KRAS*+/*TP53*+ NSCLC (10%) did not have good responses to phase 1 clinical trial therapy, had considerably worse PFS and tended to have worse OS than did those without these mutations. Patients with *KRAS* hotspot mutations at the locations other than codon G12 had markedly worse survival than did those with mutations at the codon G12 and those without *KRAS* mutations. Introduction of the EAp53 score system revealed that EAp53-LR patients with a low EAp53 score had a remarkable median OS longer than 5 years, which was significantly better than EAp53-HR patients with a high EAp53 score and those without *TP53* mutations. Our data supported that the matched phase I trial therapy had greater clinical benefit and produced better PFS than did the unmatched therapy. The association of improved clinical benefit and PFS with the antiangiogenic phase I trial therapy in patients with *TP53* hotspot mutations provided additional evidence that antiangiogenic agent-based phase I trial therapy is appropriate for consideration as matched therapy in patients with *TP53* mutant NSCLC.

## MATERIALS AND METHODS

### Patient selection

From June 2011 to December 2016, 185 consecutive patients with advanced NSCLC were referred to a phase 1 trial center at MD Anderson and underwent molecular tests for genetic aberrations in their tumors. Patient demographics, medical histories, Eastern Cooperative Oncology Group performance statuses, laboratory results, gene aberration results, and outcomes of treatment administered in the phase 1 clinical trials were obtained from their electronic medical records. In accordance with the guidelines of the MD Anderson Institutional Review Board (IRB), this study was conducted under an IRB-approved protocol with waiver of informed consent.

### Genomic hotspot mutation and variant detection

Next-generation sequencing was performed to detect somatic mutations in the coding sequences of a total of 46 or 50 genes [50] using the Ion AmpliSeq Cancer Hotspot Panel (Life Technologies) on the DNA extracted from the tumor samples in the Clinical Laboratory Improvement Amendments-certified Molecular Diagnostics Laboratory at MD Anderson as described previously [25, 51]. Genomic DNA from each sample was used for sequence analysis of hotspot mutations, including those at exons (codons) 2-3 (5-66) and 4 (114-150) of the *KRAS* gene and exons (codons) 2 (1-20), 4 (68-113), 5 (126-138), 5-6 (149-223), 7 (225-258), 8 (263-307), and 10 (332-367) of the *TP53* gene.

### The evolutionary action score system of *TP53* missense mutations

The evolutionary action scores of *TP53* missense mutations (EAp53s) were calculated based on a model of the phenotype-genotype relationship in which protein evolution was hypothesized to be a continuous and differentiable process as described previously [52–54]. The EAp53 scores ranged from 0 to 100, with higher scores representing more deleterious alterations according to an EAp53 server (http://mammoth.bcm.tmc.edu/EAp53; Baylor College of Medicine). An EAp53 threshold of 75 was selected to classify a specific mutant p53 protein as low- or high-risk (EAp53-LR and EAp53-HR, respectively) [37, 38].

### Treatment of NSCLC and survival evaluation

The decision to enroll an eligible study patient in a phase 1 clinical trial depended on protocol availability and the discretion of the treating physician. Tumor responses to phase 1 trial therapy were evaluated according to the Response Evaluation Criteria in Solid Tumors (version 1.1) [55]. All patients were followed until death or censored on May 1, 2017. Progression-free survival (PFS) was defined as the time from study entry to the date of first objective documentation of progressive disease, date of death, or censor date. Overall survival (OS) was defined as the time from the date of the first phase 1 clinical trial visit (OS-phase I) or the date of the initial metastasis diagnosis (OS-metastasis) to the date of death or the censor date, regardless whether they received a phase I trial therapy.

The phase 1 clinical trial therapy was considered matched therapy if the patient received one or more agents targeting an actionable genetic aberration or proteins downstream from it, such as an EGFR inhibitor for an EGFR mutation [56], a BRAF inhibitor or a mitogen-activated protein kinase kinase (MEK) inhibitor for a BRAF mutation [57, 58], and crizotinib for ALK or ROS1 rearrangement [10, 11].

### Statistical analyses

Continuous interval-scaled data were summarized using median values and ranges. Categorical data were summarized using frequencies and relative frequencies. Associations between categorical variables were tested using the chi-square and Fisher exact tests. PFS and OS curves were estimated using the Kaplan-Meier method and compared using log-rank tests. A second-order effect on hazard ratio (HR) was analyzed by Cox proportional hazards regression analysis through backwards conditional elimination, adjusted for with selected co-variables collected at initial phase I clinic visit (Eastern Cooperative Oncology Group performance status, albumin, lactate dehydrogenase, and the number of metastatic sites). All tests were two-sided and considered significant when *p* values were less than 0.05. Statistical analyses were performed using the SPSS software program (version 24; IBM Corporation).
